# The impact of timetable on student’s absences and performance

**DOI:** 10.1371/journal.pone.0253256

**Published:** 2021-06-25

**Authors:** Souad Larabi-Marie-Sainte, Roohi Jan, Ali Al-Matouq, Sara Alabduhadi

**Affiliations:** 1 Computer Science Department, College of Computer and Information Sciences, Prince Sultan University, Riyadh, KSA; 2 Production and Manufacture Department, College of Engineering, Prince Sultan University, Riyadh, KSA; Lingnan University, HONG KONG

## Abstract

Student’s academic performance is the point of interest for both the student and the academic institution in higher education. This performance can be affected by several factors and one of them is student absences. This is mainly due to the missed lectures and other class activities. Studies related to university timetabling investigate the different techniques and algorithms to design course timetables without analyzing the relationship between student attendance behavior and timetable design. This article first aimed at demonstrating the impact of absences and timetabling design on student’s academic performance. Secondly, this study showed that the number of absences can be caused by three main timetable design factors: namely, (1) the number of courses per semester, (2) the average number of lectures per day and (3) the average number of free timeslots per day. This was demonstrated using Educational Data Mining on a large dataset collected from Prince Sultan University. The results showed a high prediction performance reaching 92% when predicting student’s GPA based on absences and the factors related to timetabling design. High prediction performance reaching 87% was also obtained when predicting student absences based on the three timetable factors mentioned above. The results demonstrated the importance of designing course timetables in view of student absence behavior. Some suggestions were reported such as limiting the number of enrolled courses based on student’s GPA, avoiding busy and almost free days and using automated timetabling to minimize the number of predicted absences. This in turn will help in generating balanced student timetables, and thus improving student academic performance.

## 1 Introduction

Since the last decade, the Higher education is becoming vital for individuals to ensure their future life in both financial and educational aspects. Academic performance is an important element for the students in the accomplishment of the high education. It is also the achievement level the institutions need to meet in order to reach the educational goals. This knowledge can be used to identify susceptible students at risk of falling or in need of further consideration. Therefore, students’ academic performance is becoming an interesting topic leading to explore two main research directions 1) predicting the academic success and 2) investigating the factors affecting the students’ academic performance. The performance of students might be influenced by numerous factors such as course difficulty [1], e-learning resources [2], the institution teaching strategies [3], etc. However, the absenteeism is another main factor affecting students’ performance. This is because the missed classes are problematic for a student to overtake the work missed, not mentioning the lectures and other activities in class. Most state-of-the-art studies on student success stressed that class attendance is correlated to better performance [4–7].

So, what triggers student absenteeism? Some related works considered that class attendance may be regarded as student engagement that can also be influenced by other factors such as the technical and educational resources available [6]. Others demonstrated that student absenteeism is due to psychological reasons [8]. One overlooked aspect in these studies that can lead to absenteeism is the course timetabling design. University timetabling is a comprehensive educational schedule for courses that includes the time and location of classes. It has been noticed that some students have a full schedule comprising four or more consecutive lectures in a day which might become stressful leading to fatigue and skipping of classes. On the other extreme, other students may have two or three lectures per day separated by a large break which also may lead to skipping of classes.

This study looked at demonstrating that the design of the course timetable is one of the main causes of absences, and thus one of the factors affecting student academic performance. To the best of our knowledge, the impact of timetabling design on student absenteeism has not been explored. According to [9], timetabling design research has been neglected. The authors in [10] showed the preferences of students regarding their instructors and timetable but they have not investigated the relationship between timetable design and student absences.

Hence, the aim of this study is to show the impact of the course timetable design on student academic performance. To achieve this goal, three main objectives are tackled in this study:

1. Show that the absenteeism can affect the students’ performance. This is performed by showing the relationship between both variables (absences and GPA) and then predicting student’s GPA based on the absences and some factors related to timetabling design.
2. Show that the absences can be triggered by the timetabling design. This is carried out by showing the impact of some timetabling factors (such as the number of courses per semester, the average number of lectures per day, and the average number of free timeslots per day) on the absences, and then predicting the absences based on these timetable factors.
3. Help improving the design of university timetable which eventually can ease the learning process. This is realized by providing new directions to timetabling researchers.

To this intent, Educational Data Mining (EDM) was applied on a large student dataset collected from a university in Riyadh, Saudi Arabia. The data was carefully preprocessed to remove outliers. Then, a correlation metric, linear regression, and two well-known classifiers (the Neural Network and the k-Nearest Neighbor) were employed to investigate the different causal relationships hypothesized. A discussion of the results followed. The rest of the article is structured as follows. Section 2 presents the most recent related studies. Section 3 states the problem under discussion. Section 4 addresses the methodology used. Section 5 discusses the experiments and answers to the research questions. Finally, Section 6 concludes this research work.

## 2 Related work

In this section, the recent related works are presented. The work surveyed explore four main research directions. 1)- The studies that predicted the students’ academic performance. 2)- The research works that tackled the students’ performance at course level. 3)- The studies that investigated the enhancement of the educational level. 4)- Finally the studies that demonstrated part of this research’s aim including the affect of absences on the students’ performance and the affect of a disorganized timetable on the students’ attendance and then the students’ performance. The section ends with a summary showing the contribution of this research article.

The researchers were mainly based on predicting students’ performance using different classification and clustering algorithms. For example, the authors in [11] predicted students’ performance based on their university’s aspects. They applied two rule learners (JRip and OneR), two popular Bayes classifiers, a decision tree, and a Nearest Neighbor classification algorithm on a data collected from universities, composed of 10330 students (the attributes were not mentioned). The results indicated that the Bayesian classifier achieved an accuracy of 60%, and the Decision tree reached 67%, the k-NN classifier accuracy was about 60% while that of JRip and OneR classifiers were 63% and 55% respectively. However, the classifiers performed differently due to some attributes associated with the students’ University Admission Score that affected the classification. In [12], the authors measured the effect of the recommended and personalized plans on students’ performance. They aimed also at predicting the students’ GPA. They employed Jaccard coefficient on three different prediction models: Regression Tree (RT), Support Vector Regression (SVR), and Linear Regression (LR) to measure the similarity between the registered courses and the eventual planned courses. They used both RapidMiner 5.1 and WEKA software. The dataset was collected from the student records of King Abdulaziz University from 2007 to 2011 (the attributes were not mentioned). The accuracy obtained by using the RT was 0.459, the SVR was 0.44, and the LR was 0.602. Another study about predicting students’ grades [13] using the Bayesian networks framework was investigated. The dataset was collected from Illinois University of Chicago from 2003-2012. It was composed of 300 engineering students (the attributes were not mentioned). The obtained results were compared against those obtained by employing Decision Tree, the Naive Bayes and the K-nearest Neighbors algorithms. According to the authors, the above-mentioned model outperformed the other models in predicting grades. The accuracy of predicting the grades in physics course was 70.4%, in math course was 73.1%, and in another science course was 35.6%. However, the authors didn’t take into consideration the social, personal, and psychological factors which can positively affect the students’ performance. Moreover, the authors in [14] applied decision tree algorithms (J48 and C4.5), Bayesian classifiers (BayesNet and Naive Bayes), two rule learners (JRip and OneR), and Nearest Neighbor algorithm (IBk) to predict students’ performance. The authors used Weka software on in-house dataset collected by the authors from three colleges in Tamil Nadu State. The obtained results indicated that the overall classifiers reached an accuracy greater than 60%. JRip presented the highest accuracy compared to other classifiers. However, it has been recommended that various data mining techniques can be utilized on an expanded dataset to obtain more accurate results. Furthermore, in [15], students’ performance using five classification algorithms was predicted; namely Multilayer perceptron (MLP), Naïve Bayes, REP tree, and Decision tree (J48). The collected dataset was composed of 127 students and 7 attributes. The dataset was split into training and testing sets (the splitting percentage or method were not mentioned). The best result (ROC = 0.7206) was obtained by using Naïve Bayes. The authors interpreted the results as ‘A’ if the ROC is above 70%. They suggested to consider in a future work the location and the psychological state of the students because these factors might affect the students’ performance. Last but not least, in [16], the authors assessed the student performance by combining the Neural Network and Support Vector Machine with K-Means clustering technique. The authors used a dataset called “Results for State Assessments in Reading/Language, Arts and Mathematics” collected between 2008 and 2011 by the Education Project department in United State. The results indicated that the Neural Network performed better than the Support Vector Machine. The mean square error achieved an improvement of 5-20%. The authors suggested a deep investigation of the parameter setting to enhance the results. Last but not least, the authors in [17] showed that the social interaction affects the students’ academic performance. The dataset was collected from the economics department in Russian university during one academic year (2013-2014). The authors used the stochastic actor-oriented models. They modeled the evolution of the academic performance with both the friendship and the study assistance networks. The results highlighted the important affect of friendship in the pedagogical environments.

The researchers also investigated the performance at the course level. For example, the authors in [18] used several classification and clustering algorithms to predict whether the students pass a course or not. For the classification, they applied Function-based, Tree-based, Rule-based, and Bayes-based algorithms. For the clustering, they employed Sequential Information Bottleneck, Hierarchical Cluster, Simple K-Means, X-means, Expectation Maximization, and Farthest First algorithms. The dataset was collected from online discussion forums retrieved from Moodle (the size and the attributes were not mentioned). The experimental results showed that only the Expectation Maximization clustering algorithm has got a similar accuracy and F-measure to those obtained by the classification algorithms. In [19], identification of students at-risk in chemistry was studied by employing different techniques, including One-way multivariate analysis of variance (MANOVA) and One-way ANOVA. Three datasets were collected to evaluate students’ self-concept as a learner of chemistry to evaluate students’ behavior relating to chemistry courses between 2008 and 2013, and to assess students’ motivational orientations. However, the authors didn’t mention more details about the datasets collected. In their findings, the authors stated that both the behavior and the self-concept negatively affected performance of students. Another study [1] investigated the impact of a course’s difficulty level. The authors used the Classification And Regression Tree (CART) combined with AdaBoost, in addition to J48, Reptree, Nave Bayes, Multilayer perceptron (MLP), Linear Regression (SMOReg) and Non-linear regression model (MSP). They used two datasets composed of 1000 undergraduate students from Engineering and Computer Science colleges in a State university. The results indicated that both CART and J48 yielded the highest accuracy reaching 98% compared to NaiveBayes and REPTree classifiers. However, the other classifiers such that MSP yielded good results when predicting specific courses.

Enhancing the educational system was another research goal the researchers examined. For example, the authors in [2] studied the feedback related to Moodle utilization to improve the e-Learning quality and to timely deliver suitable solutions for students’ problems. The authors applied five classifiers (Naive Bayes, K-NN, Decision Tree, Random Forest, Generalized Linear Model) using real data collected during one semester. The data was preprocessed to enhance the classification accuracy. The best accuracy was obtained by Generalized Linear Model equaled to 99%. The authors stressed that the obtained results were valuable to classify and provide an immediate support to students’ problems. In [20], students’ progress was predicted to enhance the effectiveness of the educational system. The authors used several classification algorithms (J48 Tree, Bayesian Network, and Random Forest). The dataset was collected based on the students’ attendance, assessments, and marks. Then, it was divided into five classes namely Fail, Below Satisfactory, Satisfactory, Good, Very good. The authors experimented the original data (without resampling) and the resampled data. The results showed that the random forest is the most effective classifier in predicting the students’ performance while the BayesNet and NaïveBayes were the least effective ones. The study identified low level students needing special attention. This helped in reducing the students failing rate. In [3], the study improved the institution learning and teaching experience using students feedback related to lab facilities, classrooms, and exams. The authors didn’t give details about the dataset. The collected dataset was preprocessed and prepared for the classification step. In addition, feature selection was applied to extract the relevant words. The random forest algorithm was used to classify the data into positive, negative, and neutral. The neutral class resulted with the highest accuracy. In [21], the authors proposed a map of courses knowledge using three representation vector techniques (bag of words, skip-grams and CBOW) and Linear Regrassion. They aimed at discovering the relationships between the courses through their content descriptions. The course description was represented by a vector using the aforementioned representation methods. They utilized a datast from UCI composed of 124000 students enrolled in different courses. The obtained course representations revealed a high fedelity surpassing their content description and showing the differences between the analogous courses.

The aim of this research is to prove that the absences caused by a disorganized timetable could affect the students’ performance. Part of this aim was investigated by [6]. The authors demonstrated that class attendance affects the students’ academic performance. However, the study was based on the geographical location of classes and their attendance rate. The collected data was composed of 1000 undergraduate students. The geographical location was calculated using Bluetooth and GPS. The authors used a statistical analysis based on the correlation and the relationship between attendance and some factors like the grades. The results showed that the failing rate can highly be caused by the low attendance level. The authors revealed some limitations such that the noise in detecting the class location that affected the calculation of the distance. Besides, the students who took part in this study are far from the average students as they have high grades. Another part of this research’s aim was studied by [10]. The authors aimed at enhancing the timetable because it was claimed to be one main factor in enhancing the students’ performance. The students were involved in the choice of timing, instructors, and material, etc. The authors used a questionnaire to collect the dataset which was composed of 38 attributes. The attributes consisted of the enrolled students, their gender, educational background, area, etc. The authors used k-Means and a priori clustering algorithms in addition to association rules to show the tendencies of students regarding their instructors and the timetable. The results determined some students’ preferences such as open book exams, 60 min class timing, mixed language, etc.

To sum up, many studies were published to study the students’ performance or enhancing the educational system. All the presented studies were performed on collected and private datasets. The details mentioned about these datasets (such as the size and the attributes) are insufficient. In addition, no comparison was done because of unavailability of the datasets. The authors in most of these studies aimed at applying different Data Mining (DM) algorithms for the purpose of algorithm performance comparison. Only two studies tackled timetabling and class attendance. The first one [10] investigated student preferences in the design of the course timetable in terms of instructors, timing, facilities, etc. but did not study the effect of the timetable design on student absentees. The second study [6] investigated the geographical location of classes and the attendance rate. Hence, the present study is the first to investigate students’ performance based on the absences caused by the timetable design.

## 3 Problem statement

Absences can negatively affect student academic performance because of missed classes that could be very difficult to retake or compensate for. In addition, the student may lose the opportunity to make up missed course assessments. It has been observed that the absences can be influenced by students’ course timetable design. In fact, a large number of courses can increase the number of lectures per day which makes busy and stressful days for students. This can provoke students missing some lectures during a day. On the other hand, the number of free timeslots (breaks) per day can be either large or null. This study demonstrates that both cases can lead to students missing lectures. Last but not least, it is assumed that the number of free days may affect the students’ attendance.

Hence, seven research questions are addressed.

1. Can the student’ performance (student’s GPA) be affected by the absences?
2. Can the GPA be predicted based on the absences and the factors causing the absences?
3. Can the absences be caused by the number of courses assigned to students during one semester?
4. Can the absences be caused by the average number of lectures per day?
5. Can the absences be caused by average number of breaks per day?
6. Can the absences be caused by the number of off days per week?
7. Can the absences be caused by the above factors?

**Fig 1:**
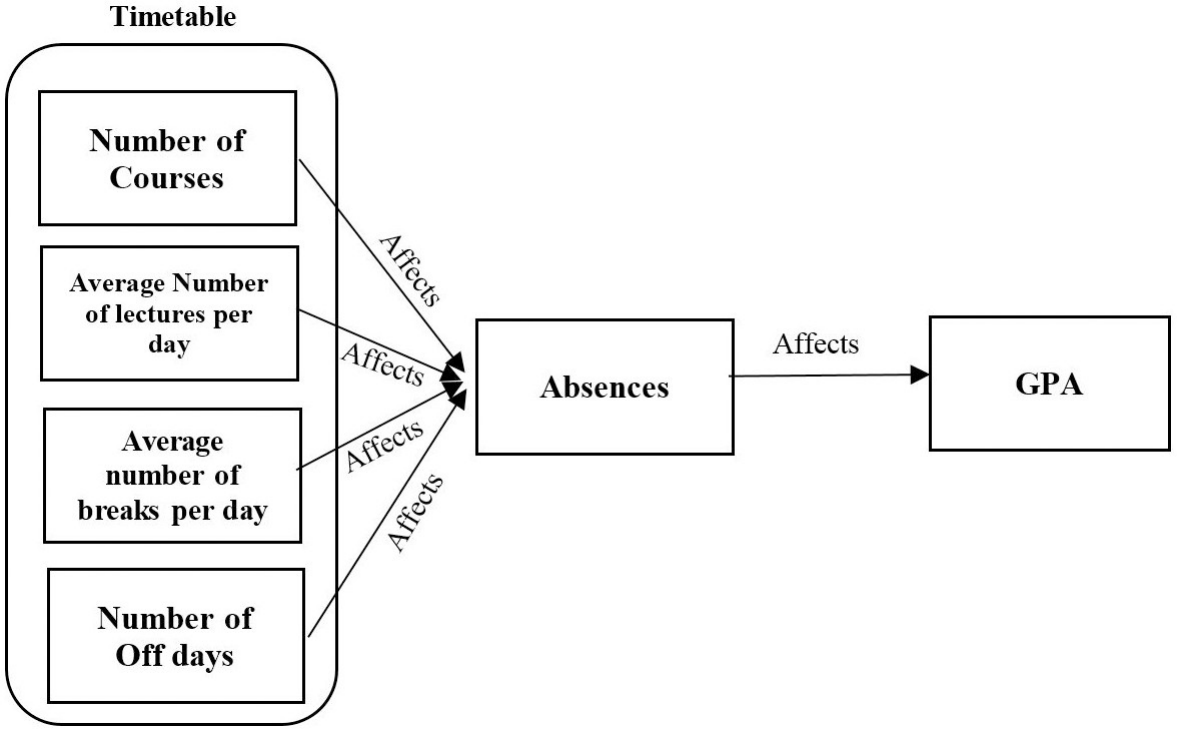
Impact on student’s GPA by the number of students’ absences due to timetabling factors.

Finally, the aim of the present work is to demonstrate that the GPA can be affected by the factors of student’s timetable as indicated in Fig 1.

## 4 Methodology

In order to answer the research questions, three main phases were discussed. Correlation was employed to indicate the strength of the relationship between two variables. Then, Regression was applied to show whether the proposed hypothesizes (the research questions) are significant and can be supported or not. Finally, the validated models were used to predict the GPA and the absences.

### 4.1 Correlation

First of all, the correlation coefficient is considered to indicate the strength of the association for two variables. This coefficient is between -1 and 1. If this coefficient is less than -0.2 or greater than 0.2, than the relationship between both variables is strong. In this study, the correlation coefficient was calculated between the dependent variable (GPA) and the explanatory variable (the number of absences). It is also calculated between the absences and each of the timetable factors mentioned in the previous section. In this study, the Spearman Correlation is used. It is defined as:

$$\begin{array}{c} Cor=\frac{\sum \left(x^{\prime }-m_{x}\right)\left(y^{\prime }-m_{y}\right)}{\sqrt{\sum {\left(x^{\prime }-m_{x}\right)}^{2}\sum {\left(y^{\prime }-m_{y}\right)}^{2}}} \end{array}$$

Where *x*′ = *rank*(*x*) and *y*′ = *rank*(*y*), *m*_*x*_ and *m*_*y*_ are the means of *x* and *y* respectively.

### 4.2 Regression

The Main method used in this study was the Linear Regression to show whether the proposed hypothesizes is significant or not. The Linear Regression predicts the response variable based on the independent variable. If there are more than one independent variables, this method is named Multiple Linear Regression. This method provides an equation of the form:

$$\begin{array}{c} \hat{Y}=B_{0}+B_{1}X_{1}+B_{2}X_{2}+\ldots +B_{m}X_{m} \end{array}$$

Where (*X*_1_, *X*_2_, …, *X*_*m*_) are the independent (explanatory) variables represented by vectors of size *n* as follows: *X*_*ij*_ = (*x*_*i*1_, *x*_*i*2_, …, *x*_*in*_) where *i* = 1, …, *m*, *m* is the number of independent variables in the model, *n* is the sample size.

$\hat{Y}=\left({\hat{y}}_{1},{\hat{y}}_{2},\ldots ,{\hat{y}}_{n}\right)$ is the vector of the dependent variables, where ${\hat{y}}_{i},i=1,\ldots ,n$ be the predicted value of the dependent variable for the *i*^*th*^ observation

*B*_0_ = (*b*_01_, *b*_02_, …, *b*_0*n*_) be the intercept (constant).

(*B*_1_, *B*_2_, …, *B*_*n*_) are the regression coefficients of the independent variables, defined as *B*_*ij*_ = (*b*_*i*1_, *b*_*i*2_, …, *b*_*in*_) where *i* = 1, …, *m*.

The main result provided by the Linear Regression is the p-Value of the intercept and the coefficients of the explanatory variables. The p-Value indicates if the assumed hypothesis is rejected or not. In other words, the statistical model is supposed to be significant if the obtained p-Values are less than the significance level (in this study, it is equal to 0.05). In addition, several other measures can be considered.

The F-statistic determines how well the linear regression model fits the dataset. The higher the F-statistic the better it is.

The residual standard deviation is another measure that shows the difference between the predicted and observed values. The smaller this measure the better it is.

The R Square (also called coefficient of determination) describes the global relationship between the dependent and independent variables. It constitutes the square of the multiple correlation coefficient. It is the best measure to asses the goodness of fit. For more details, one can refer to [22]

### 4.3 Prediction

In this research, two main predictions were performed. The first one is prediction of the GPA based on the absences. The second is the prediction of the absences based on the timetable factors (discussed in the above section). To this end, two well-known classifiers were employed; namely the k-Nearest Neighbors and the Neural Network. To validate the prediction results, several evaluation metrics were calculated: the accuracy, the precision, the recall, the F1 measure, the specificity and the sensitivity.

In the second prediction, two classes were created (discussed in experiment 4). These classes were unbalanced forming a majority and minority classes due to the unbalanced datasets used in this study.

- Majority class: comprises many instances from the dataset where the prediction is easily performed.
- Minority class: comprises few instances from the dataset where the prediction is difficult or impossible to be done.

**Fig 2:**
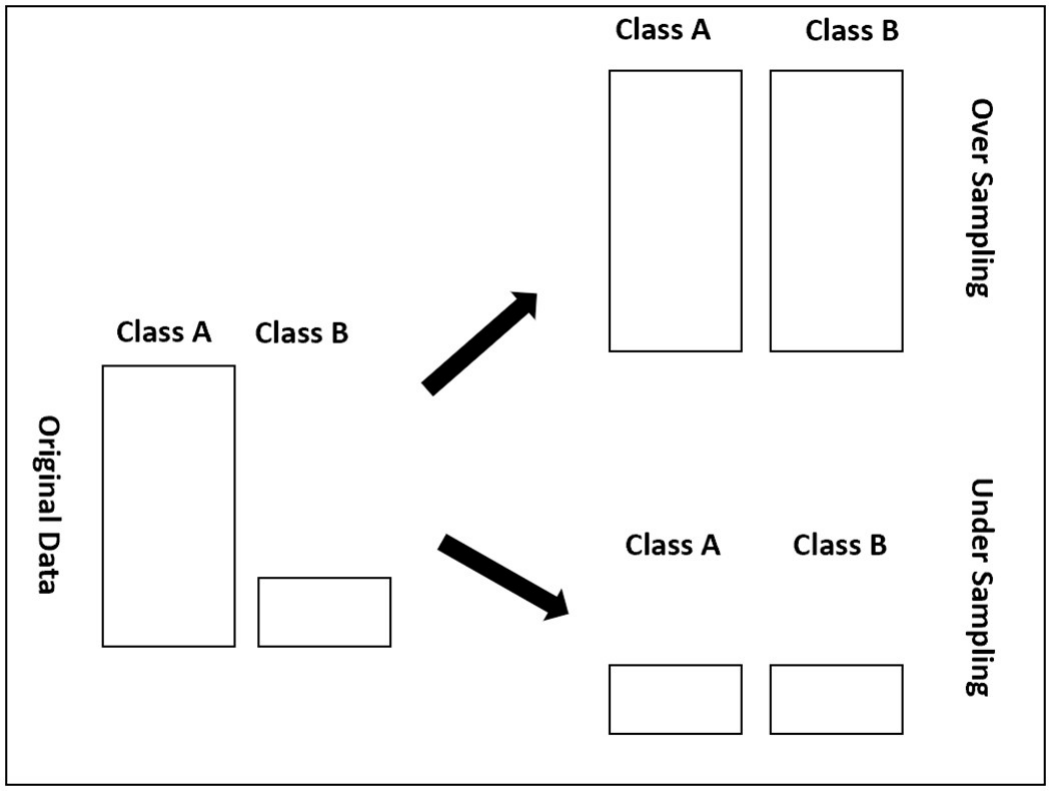
Representation of Over and Under Sampling.

Unbalanced dataset comprises unequal instances for different classes which can result into classification problems. This is because most classifiers are susceptible to unbalance in the majority classes. In fact, only the more common class (majority class) will be predicted which makes the prediction biased [23]. The minority class is usually the more important class that requires investigation and hence improving its recognition is important [24]. To do that, it is necessary to balance the datasets using some sampling technique. In this article four sampling methods were used as follows:

1. Over-Sampling: consists of randomly reproducing samples or creating other instances based on the data to make the classes with fewer instances significant. Fig 2 depicts this representation.
2. Under-Sampling: involves randomly selecting a set of instances from the largest class to fulfill the number of instances originating from minority class as depicted in Fig 2
3. Rose Sampling: generates artificial balanced instances using a smoothed bootstrap technique. This sampling technique handles binary classification problems with unbalanced classes [25]. The process of ROSE for generating a new artificial instance (case) is explained in Algorithm 1
4. Synthetic Minority Over-Sampling Technique (Smote): produces synthetic instances rather than duplicating them to enlarge the minority class [26]. The new instances are inputted in the minority class using the k-Nearest Neighbors where the neighbors are randomly selected. The SMOTE technique was clearly explained in the Algorithm 2

#### 4.3.1 Neural Network

Neural Network (NN) is a supervised Machine Learning algorithm that imitates the human brain process, developed for the first time in 1943 [27]. NN is a connected graph with artificial neurons (also called nodes) and weighted edges (also called connections). The neurons receive and transfer input/output data through the weighted edges using a mathematical function called activation function. The neurons are aggregated into layers. The first layer (called the Input layer) collects the data. Then, many hidden layers could be created (the simplest NN contains one Hidden Layer). The hidden layers set and adjust the weights and select the relevant features from the data for classification/prediction purpose. Finally, the Output layer involves the predicted output. NN is trained using an optimization method to find the best weights and learning rates while reducing the losses and increasing the accuracy. Usually, a gradient based learning algorithm, such as the back propagation or some variant are used.

In this study, the NN constitutes one hidden layer. The optimization method used is one variant of Quasi-Newton method called Broyden–Fletcher–Goldfarb–Shanno algorithm (BFGS) since it is known to be efficient in the optimization of complex problems [28]. Moreover, the logistic activation function is employed. The main parameters that require tuning are the weights and the size of the network. To update the weights, the weight decay was used which is one parameter in the weight update rule that leads the weights to exponentially decrease to zero, if no other method is scheduled for updating the weights [29, 30].

**Fig 3:**
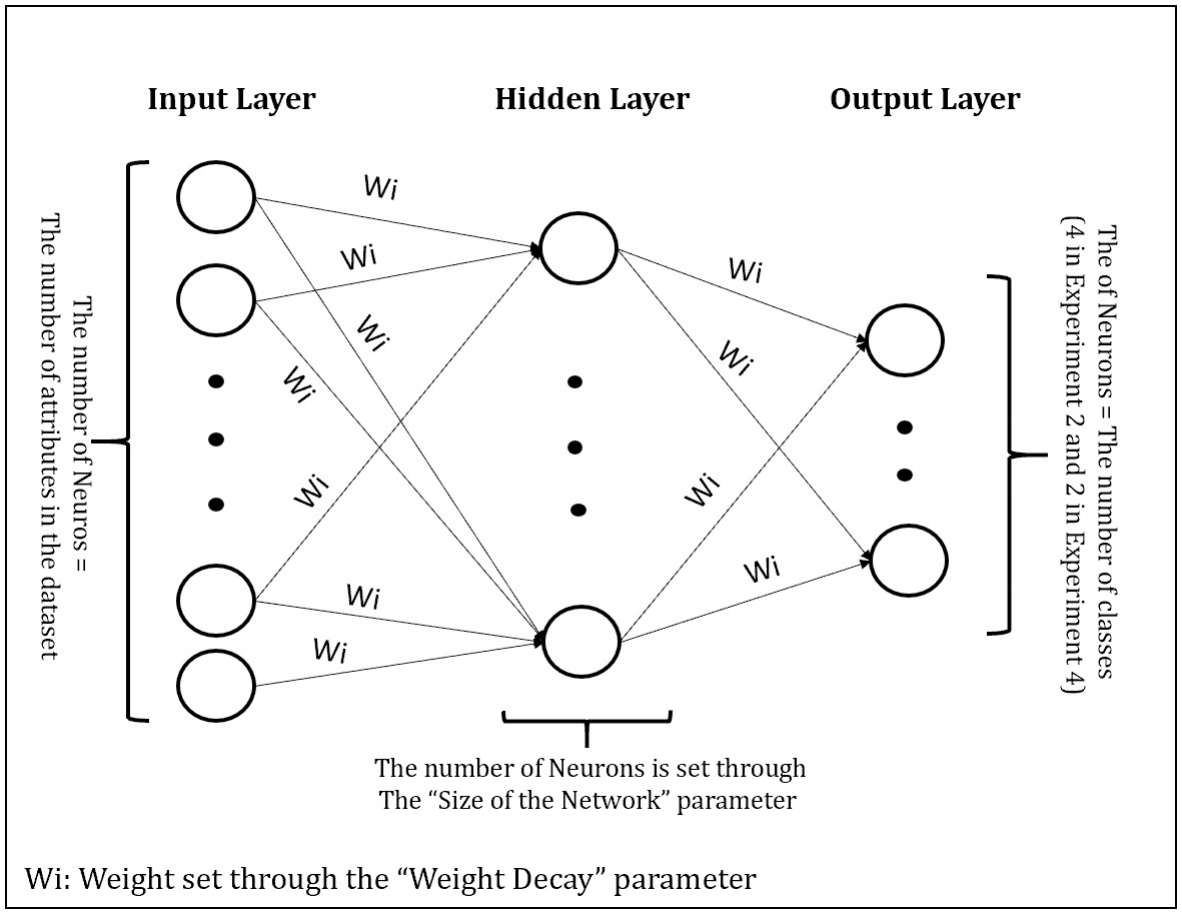
The architecture of the Neural Network used.

The size of the Network is the parameter that controls the architecture of the network. It consists of the number of neurons in each hidden layer (in this study one hidden layer was used). Specifying this number is a must when establishing the network. Many studies were published in this regard, for example [31]. It is worth to mention that the number of neurons in the input layer is equal to the number of attributes in the dataset. Whereas the number of neurons in the output layer is the number of classes in the classification problem. Fig 3 displays the Neural Network used in this work. Both parameters (the size of the network and the weight decay) are studied and set in Experiment 2.

#### 4.3.2 K-Nearest Neighbors

K-Nearest Neighbors (k-NN) is a supervised machine learning technique [32] known by its simplicity and efficiency in tackling complex problems. k-NN is based on a similarity measure. The Classification of a case is performed using the majority poll of its “k” neighbors. The best value of k is set based on the best accuracy. The similarity measures could be the Euclidean distance, Manhattan distance, Normalization, Z-score normalization, etc. In this study, the Euclidean distance was employed.

The Euclidean distance consists of the length between two data points. Let suppose that the dataset contains *N* attributes, then this distance between two data points *x* = (*x*_1_, *x*_2_, …, *x*_*N*_) and *y* = (*y*_1_, *y*_2_, …, *y*_*N*_) is defined as follows.

$$\begin{array}{c} d\left(x,y\right)=\sqrt{\sum_{i=1}^{N}{\left(x_{i}-y_{i}\right)}^{2}} \end{array}$$

## 5 Experimental results

This section includes data collection, data preprocessing, and many experiments replying to the seven research questions. The experiments were performed using Dell XPS 9343 with an Intel^®^ core TM i7-5500U CPU 2.40 GHz and 8-GB RAM. In addition, R version 3.5.2 was utilized for Data Mining techniques.

### 5.1 Data collection and preprocessing

The dataset was collected from Prince Sultan University, a private University in Saudi Arabia. It consists of relevant information extracted from the timetables of two main colleges, the Engineering College (ENG) and the College of Computer and Information Sciences (CCIS) for three consecutive academic years (from 2016/2017 until 2018/2019). Both Colleges have three departments making the study on 6 different college programs. Ethical approval was obtained from the Institutional Review Board (IRB) of Prince Sultan University to approve collecting student data from university databases for research purposes. All data was made anonymous according to IRB standards and policies. The dataset was preprocessed in order to keep the relevant attributes needed in this study and remove the seldom cases that can represent outliers. There are 11 attributes and 4325 instances. The attributes are defined in Table 1. In this dataset, the instance represents a student. The number of instances (students) in each college for these three academic years are indicated in Table 2.

**Table 1:** The dataset attributes and their descriptions.

Attribute number	Attribute name	Description
1	ID	Student Identification Number.
2	Prev GPA	GPA of the previous semester.
3	GPA	GPA of the current semester.
4	Department	Department to which student belongs to.
5	Level	The academic level of a student such as Freshman, Sophomore, Junior and Senior.
6	Term	Academic Term. Five academic terms were considered in this study (fall and spring terms between 2016 and 2019).
7	Attempted_Hours	Number of registered hours in a semester per student.
8	Number of courses	Number of courses enrolled by a student.
9	Number of Off days	Number of days when there are no classes for a student.
10	Average Lecture per day	Average number of lectures per day.
11	Average break per day	Average number of hours students are having a break.
12	Total Number of absences	Total Number of absences for a student throughout the semester.

**Table 2:** The size of CCIS and ENG datasets.

Datasetr	Sample Size
ENG	2661
CCIS	1664
Total	4325

### 5.2 Experiment 1

Can the student performance (student’s GPA) be affected by the absences?

The first experiment consists of showing that the absences negatively affect the students’ performance using the Correlation and Linear Regression.

#### 5.2.1 Correlation

**Fig 4:**
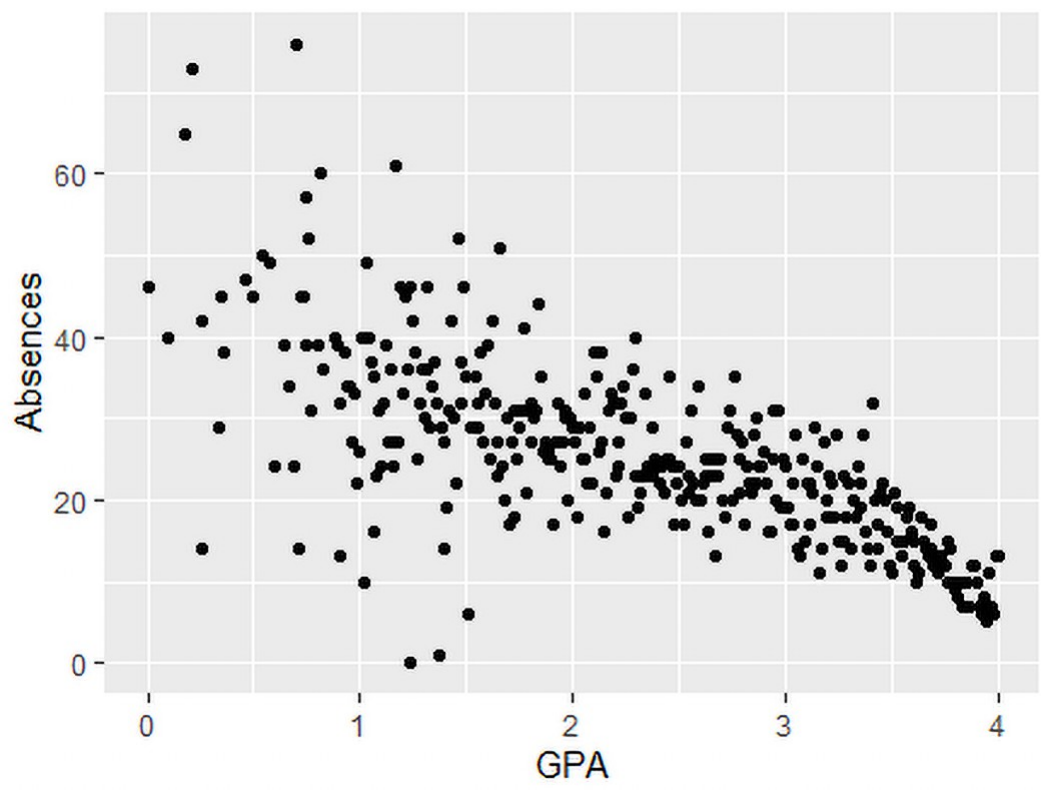
Scatter plot of the average number of absences and the GPA.

The Correlation between the average number of absences and the GPA for the whole dataset was found to be equal to −0.7052 which means that when the response variable (the GPA) increases, the predictor variable decreases (the average number of absences) and vice versa. Fig 4 shows a plot of the response variable (GPA) versus the predictor variable (Average number of absences) demonstrating a strong linear correlation (strong relationship).

#### 5.2.2 Regression

The Linear Regression was applied to the whole dataset to establish the relationship between the response (GPA) and the predictor variable (the average number of absences named Abs). The confidence level is set to 0.05%. The mathematical model obtained are as follows:

*GPA* = 3.89056 − 0.06265*Abs*

The result of this prediction was validated by means of the correlation accuracy (calculated between the dependent and independent variables) reaching 0.686. As seen, this predicted results is strongly correlated with the actual values which means that the model is capable of predicting new results based on the actual data.

The *p*–*Value* for the predictor variable is less than the confidence level 0.05, as indicated in Table 3 (column 4). Thus, the null hypothesis is rejected even through the coefficient of Abs is small (−0.06265). Table 3 also displays the standard error values that are relatively small. The F-statistic is equal to 239 and the R-square equals to 0.471 which show the fit of this linear regression model to the dataset. In other words, half of the output (predicted GPA values) is explained by the model’s inputs (the Absence variable).

**Table 3:** The results of the Linear Regression for the GPA variable using the whole dataset.

	Estimate	Std. Error	Pr(> |t|)
**Intercept**	3.85732	0.11131	<2*e* − 16
**Absences**	-0.06285	0.00406	<2*e* − 16
**F-statistic**	239		
**R square**	0.471		

Consequently, this model is statistically significant and then the prediction of the GPA based on the absences is meaningful. It is worthy to say that the absences can still significantly predict the GPA even when the affect is not large (the coefficient of Abs is small −0.06265).

### 5.3 Experiment 2

Can the GPA be predicted based on the absences and the factors affecting the absences?

This experiment consists of predicting the GPA using the Neural Network (NN) and the k-Nearest Neighbors (k-NN) classifiers. To make the model more robust, the Prev_GPA variable was introduced as a new attribute. Note that, GPA and Prev_GPA (or previous GPA) are obtained for two consecutive semesters. For example, if GPA is obtained in the second semester of the academic year x, then Prev_GPA has been achieved in the first semester of the same academic year.

The whole dataset was used (4325 cases) with 7 attributes (Prev_GPA, Attempted Hours, Number of Courses, Number of Off days, Average Lecture per day, Average Break per day, Number of Absences).

The idea is to demonstrate that the factors affecting the absences can also affect the GPA. To this end, four classes are created as follows. VeryLow if the GPA is between [0, 1] (with 1 excluded), Low if the GPA is between [1, 2] (with 2 excluded), Average if the GPA is between [2, 3] (with 3 excluded), and High if the GPA is between [3, 4].

To achieve a high accuracy, the parameters of k-NN and NN classifiers were investigated. Ten-fold cross validation was applied to a training set (70% from the whole dataset), repeated 3 times. After selecting the best parameters of each classifier, the obtained model was utilized for the testing set (30% from the whole dataset).

#### 5.3.1 The Neural Network

NN Classifier has many parameters, the decay and the size of the network are most relevant ones (see the Methodology section, especially Fig 3). The experiment started by varying the decay between 0 and 1 and the size of the network between 1 and 50 based on the recommendations of [30, 31]. Only one parameter is varying at a time. After performing many trials, good results were obtained with three values for the decay 0.2, 0.5, 0.7; and four values for the size of the Network 1, 5, 10, 15. Table 4 displays the results of the accuracy (using the training set) for each combination of the decay and size values. The optimal model was obtained based on the largest accuracy value. The largest accuracy value in this experiment was 0.918 when the size values were 10 and 15. The final values used for the model are decay = 0.5 and size = 10, considering the lowest value of size (highlighted in Table 4). The optimal model was utilized in the testing stage. The results are displayed in Table 5.

**Table 4:** Parameter setting for the Neural Network.

Decay	Size	Accuracy
0.2	1	0.890
0.2	5	0.918
0.2	10	0.916
0.2	15	0.911
0.5	1	0.885
0.5	5	0.917
0.5	10	0.918
0.5	15	0.916
0.7	1	0.883
0.7	5	0.917
0.7	10	0.917
0.7	15	0.918

**Table 5:** Experimental results using both classifiers for the testing dataset.

Measures/Classifiers	NN	k-NN
**Accuracy**	0.923	0.834
**Kappa**	0.883	0.745
**P-Value**	<2*e* − 16	<2*e* − 16

#### 5.3.2 The k-Nearest Neighbors

For this classifier, the k parameter was investigated using different values. After performing many trials and based on the recommendations of [33], the values of k providing better results were between 10 to 200. The optimal model was obtained based on the largest accuracy when k = 25. This final model is utilized for the testing set, the prediction results are shown in Table 5.

To sum up, the accuracy and the kappa values for the testing dataset obtained using NN are 0,923 and 0.883 respectively (0.834 and 0.745 for k-NN respectively). So, the performance of the prediction is high. In other words, the error rates to predict the GPA using NN and k-NN are insignificant, reaching 0.077 and 0.166 respectively. The proposed models provided efficient results. In addition, the P-Value is less than the confidence level (= 0.05) which confirms that both models (k-NN and NN) are statistically significant. Consequently, the prediction results of the GPA based on the absences and the aforementioned factors are satisfactory. These relevant attributes along with the number of absences create a huge impact on the GPA.

### 5.4 Experiment 3

Can the absences be triggered by some factors related to the student’s timetable?

The aim of this experiment is to demonstrate that the absences are caused by some important factors related to the design of the timetable. To do this, the dataset of each college was treated separately because the timetable is college dependent.

The average number of absences was calculated for each college’s dataset, and then the Linear Regression was employed considering the average number of absences as dependent variable and the selected factor (the number of courses, the number of off days, the average number of lecture per day, and the average break per day) as an independent variable. The four factors were investigated independently.

#### 5.4.1 Linear Regression

Table 6 displays the p-Values and the Correlation results of the Linear Regression applied to both datasets (CCIS and ENG). The p-Values for the number of courses, the average number of lectures per day, and the average break per day for CCIS (see column 2) and ENG (see column 4) datasets are less than the confidence level (= 0.05). Consequently, our hypothesis (The absences could be triggered by some factors related to the student’s timetable) is validated for these three factors (the number of courses, the average number of lectures per day, and the average number of breaks per day). This implies that the model with these three factors is statistically significant. To confirm this result, the correlation measure of the average number of absences and each factor was calculated. As indicated in Table 6 (columns 3 and 5 for CCIS and ENG respectively), the correlation coefficient values are high which show the existence of a strong relationship between the absences and each of these factors. Although, the correlation is high for the number of off days and the average number of absences, the P-value is not met (greater than the confidence level of 0.05). Thus, the number of off days does not cause the absence (the hypothesis is not validated).

**Table 6:** Linear Regression results for the absence variable using ENG and CCIS datasets.

	CCIS		ENG	
Factors	P-Value of Coefficient	Correlation	P-Value of Coefficient	Correlation
**Number of Courses**	0.0149	0.77	0.000978	0.87
**Number of Off days**	0.0955	-0.81	0.333	-0.87
**Average Lecture Per Day**	0.029175	0.40	1.18*e* − 05	0.70
**Average Break per Day**	0.00949	0.37	0.0497	0.40

Moreover, it has been observed that when the number of courses (reaches the maximum of 9) and the number of lectures per day increases, the number of absences (also the average number of absences) increases. Similarly, when the average break per day exceedingly augments, the absences also increase. This happens when the timetable contains very busy days or when there is a very large break between lectures per day. Consequently, the theory being investigated is significant and proved for both datasets (both colleges).

### 5.5 Experiment 4

Can the absences be predicted using the above model?

In this experiment, the absences were predicted based on the above obtained model. Moreover, as the GPA and the absences have a strong relationship (see Experiment 1), it is added to the model to predict the absences. It makes sense to say that if the student had a low GPA (in the previous semester), and has a large number of courses with busy days of lectures and a minimum number of breaks per day, this can lead to absences. So, the proposed model includes the Prev_GPA (GPA of the previous semester), the number of courses, the average lecture per day, and the average break per day to predict students’ absences.

For this purpose, a new attribute was created involving two classes DN (Denial Notice) and No_DN (No Denial Notice). This idea was derived from the university’s regulation stating that a student can be denied from attending the lectures or an exam of a course if he/she exceeds a certain number of absences during the semester. This threshold was set according to the course’s credit hours. Alike the previous experiment, both datasets for both colleges are used. Since a DN is a seldom case, the datasets are particularly unbalanced. This experiment was performed as follows. Firstly, four sampling methods, as explained in the methodology section, were used to make the data balanced. Secondly, two classifiers were utilized to predict both absence classes. Finally, the results obtained using the 4 sampling methods along with both classifiers for both datasets were compared. The best model was suggested to the university to predict the absences in order to prevent any Denial case.

#### 5.5.1 Phase 1

**Fig 5:**
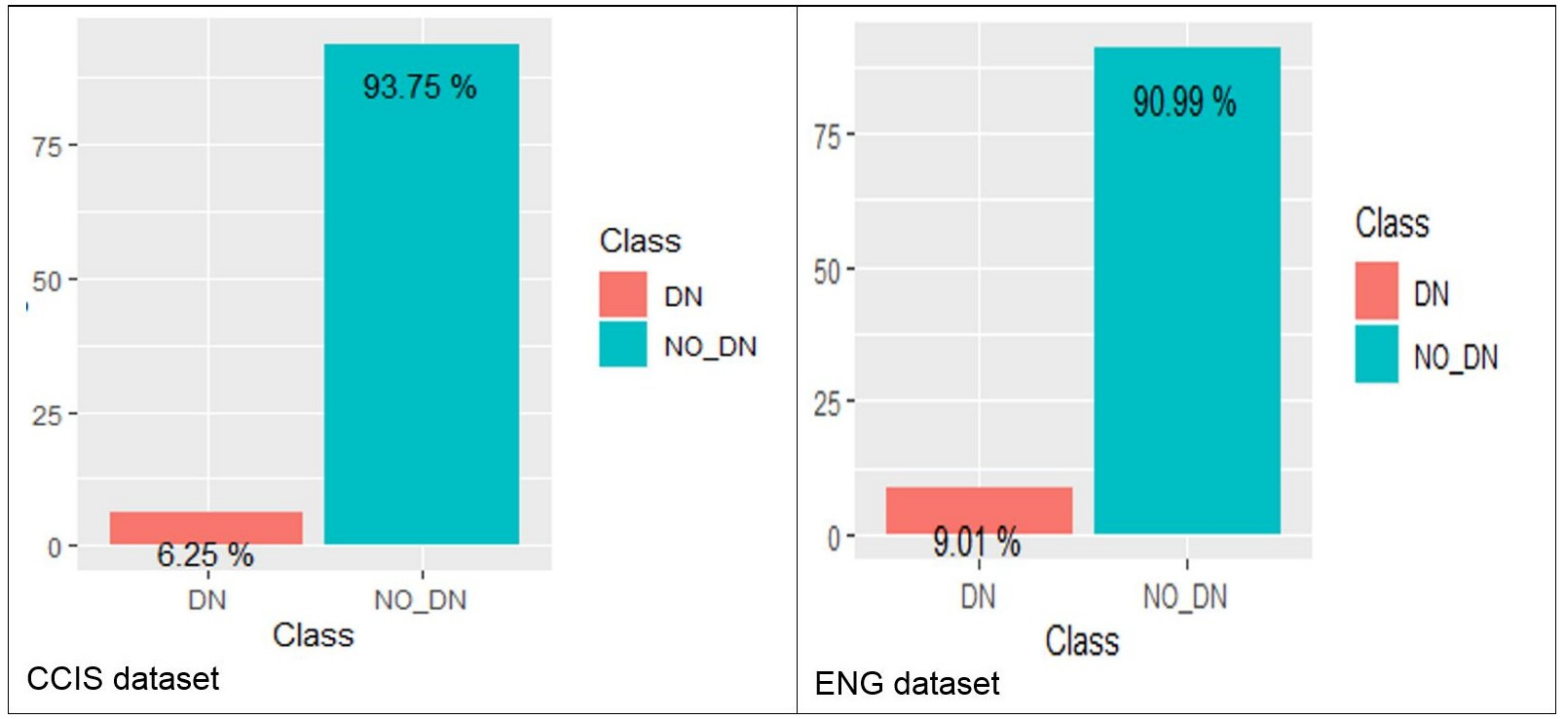
The percentage of DN and NO_DN classes for CCIS (left) and ENG (right) datasets.

As indicated in Table 7 and Fig 5, the DN class is very insignificant compared to the other class. After splitting each dataset into training (70%) and testing (30%), the sampling methods were applied to the training set.

**Table 7:** The class size of ENG and CCIS datasets before applying the sampling approaches.

Classes/ Dataset	ENG Data	CCIS Data
**No_DN Class**	2271	1560
**DN Class**	225	104
**Total**	2496	1664

#### 5.5.2 Phase 2

After balancing the training set, the classifiers were used to predict the absences. The process is similar experiment 2. The ten-fold cross validation is utilized on the training set, repeated three times, to determine the best parameters for both NN and k-NN classifiers. After the best model was found, the testing set was utilized to validate the performance of the prediction model. Table 8 shows the prediction accuracy obtained using both classifiers after applying four sampling methods to ENG and CCIS datasets.

**Table 8:** The prediction accuracy obtained using NN and k-NN classifiers to ENG and CCIS datasets.

Sampling technique	ENG		CCIS	
	NN	k-NN	NN	k-NN
**Over**	0.8316	0.6885	0.8717	0.7515
**Under**	0.7848	0.8636	0.7615	0.8076
**Rose**	0.6845	0.7206	0.7054	0.7174
**Smote**	0.7139	0.7326	0.7816	0.8657

As displayed in Table 8, both classifiers produced high prediction accuracy values using the four sampling methods for both datasets. The best prediction accuracy exceeds 80% which reflects good prediction performance. Hence, the aforementioned attributes contribute in predicting the GPA. The error rate reached an average of 0.14185, which is very low. Consequently, the absences could be predicted using the proposed model. Let explain how the highest results are obtained.

The best accuracy values yielded by NN (0.8316 for ENG and 0.8717 for CCIS) were provided by the Over-Sampling method for both datasets. This is because the Over-Sampling method increases the minority class to achieve the majority class size which makes the Neural Network more powerful as it performs well in large dataset. On the other hand, k-NN yielded the best accuracy using Under-Sampling technique for ENG dataset and Smote technique for CCIS dataset (0.8636 for ENG and 0.8657 for CCIS). The results are outstanding because the Under-Sampling method reduces the Majority class to reach the minority class’s size which diminishes the dataset size. This boosts the k-Nearest Neighbors classifier’s performance as it operates better in small dataset [15]. For CCIS dataset, this classifier obtained the best results with Smote-Sampling because this technique is based on k-NN to balance the instances throughout the classes (as explained in the methodology section) [26].

After finding the best model achieving the highest accuracy for ENG (using Over with NN and Under with k-NN) and CCIS (using Over with NN and Smote with k-NN) datasets, several prediction (classification) measures are computed on the testing set to validate the obtained results since it not possible to perform a comparison study with the state of the art works.

Tables 9 and 10 show the precision, the recall, and the F1 measure for both classes, as well as the sensitivity, the specificity, and the running time for each classifier using ENG and CCIS datasets. As specified in these tables, the precision, recall, and F1 measure values for NO_DN class using k-NN and NN are more or less than 90% which means that most of the instances belonging to that class are correctly predicted. However, for the DN class, these measures reach a value between 21% and 58% which signifies that not all the instances belonging to the DN class are correctly classified but the result remains acceptable. Moreover, the specificity values given by both classification algorithms for both datasets signify that both proposed models successfully identify the students with NO_DN case. However, the sensitivity values determine that the models can eventually detect the students with the DN case (with an average of 40%). Concerning the experiment time, the k-NN is faster than the NN due to the structure of the classifier and the fact that NN requires large dataset contrary to k-NN. Consequently, the absences can be predicted using the GPA, the number of courses, the average number of lectures per day, and the average number of breaks per day.

**Table 9:** The prediction results obtained using both classifiers for ENG dataset.

ENG /	Neural Network			k-Nearest Neighbors		
Class	Precision	Recall	F1 measure	Precision	Recall	F1 measure
**NO_DN**	0.8561	0.9542	0.9025	0.9060	0.9420	0.9237
**DN**	0.5821	0.2847	0.3824	0.4328	0.3118	0.3625
**Average**	0.7191	0.6194	0.6424	0.6694	0.6269	0.6430
**Sensitivity**	0.5821			0.4328		
**Specificity**	0.8561			0.9060		
**Exp. Time**	4.927 min			40.863 sec		

**Table 10:** The prediction results obtained using both classifiers for CCIS dataset.

CCIS /	Neural Network			k-Nearest Neighbors		
Class	Precision	Recall	F1 measure	Precision	Recall	F1 measure
**NO_DN**	0.8932	0.9676	0.9289	0.8953	0.9588	0.9260
**DN**	0.5484	0.2537	0.3469	0.4193	0.2097	0.2796
**Average**	0.7207	0.6107	0.6379	0.6573	0.5842	0.6028
**Sensitivity**	0.54839			0.41935		
**Specificity**	0.89316			0.89530		
**Exp. Time**	5.601 min			2.685 min		

## 6 Discussion

In this study, four experiments were presented. The first one consisted of showing that the GPA is affected by the absences using the linear regression.

The second experiment involved predicting the GPA based on 7 attributes (Prev_GPA, Attempted Hours, Number of Courses, Number of Off days, Average Lecture per day, Average Break per day, Number of Absences). Two classifiers (the Neural Network and the k-Nearest Neighbors) were applied to the whole dataset to predict student’s GPA. The results showed a high accuracy for both classifiers, however, NN outperforms k-NN.

Next in the third experiment, it has been shown that three timetable factors (the number of courses, the average number of lectures per day, and the average number of breaks per day) affect the absences (or the average number of absences). This assumption has been proved using two large datasets collected from the CCIS and Engineering Colleges. The Prev_GPA was used in this experiment as an attribute to enhance the prediction performance.

Finally in the last experiment, we predicted the absences based on the number of courses, the average number of lectures per day, the average breaks per day, and also the Prev_GPA using NN and k-NN classifiers. The reason of using the Prev_GPA is to give more information to the classifiers as the GPA has a strong relationship with the absences. After balancing the dataset based on DN and NO_DN classes using four sampling methods. The NN classifier performs well with the Over_Sampling method achieving an accuracy of 0.83 and 0.87 for ENG and CCIS datasets respectively. The k-NN classifier obtained a higher accuracy of 0.86 with the Under_Sampling method for ENG dataset and 0.87 with SMOTE_Sampling method for CCIS dataset. In fact, the NN classifier performs well with large dataset (provided by Over_Sampling) unlike the k-NN classifier that works well with small dataset [34]. After selecting the best models (reaching the best accuracy), the precision, recall, F1 measure, specificity, sensitivity, and running time were computed. The results showed that the NO_DN class achieved high results (exceeding 80%) with all the measures which indicates that this class is successfully predicted. However, for the DN class, the prediction achieved a maximum of 58% for ENG dataset and 55% for CCIS dataset (see the sensitivity value in Tables 9 and 10). For the experimental time, both classifiers are fast (a maximum of 5 min) but the k-NN is the fastest.

To summarize, this study successfully proved that student GPA is moderately affected by the average number of absences. Also, GPA can be predicted based on the absences and other important attributes, including previous semester GPA. The study also demonstrated through the linear regression, NN and k-NN classifiers that the absences can be triggered by the number of courses assigned to students during one semester, the average number of lectures per day, the average number of breaks per day. However, the number of off days per week is not a significant factor. The study also demonstrated that previous semester GPA has a strong influence on enhancing the prediction performance.

Finally, it is essential to clarify how the absences can affect the GPA by addressing the following questions.

1)- How the grades are assigned?

At Prince Sultan University (PSU), the grades are assigned based on the assessments approved by the department and are mentioned in the course specification. The grades are divided into midterm grades (60%) and final exam grades (40%). Midterm grades include mostly the quizzes, midterm exam, assignments, lab work, and project work, whatever is applicable depending on the nature of the course. Student attendance is not considered in course grades, which means absence from the classes will not affect directly the students’ grades. However if a student misses classes, his/her learning due to the missed lectures, class discussion, tutorials, or lab work is affected to some extent, which in reality is not compensated by simply reading the lecture notes or any learning resources excluding exceptional circumstances. As a result, the absent student is not confident enough to answer the critical thinking questions in various types of assessment aligned with bloom’s taxonomy.

2)- Are the exams based primarily on what is presented in class?

The concepts taught during the face-to-face interaction in the class are based on using varied teaching strategies such as lectures, class discussion, flipped classroom, tutorials or labs, where the students are taught how to use the knowledge in real applications meeting the 21st century skills. In addition to that, students learn to work in a collaborative environment. Such kind of learning is affected when the students are absent from the classes. They are not able to fully participate in class discussion due to the lack of knowledge from previous lectures. The exams following the bloom’s taxonomy include questions based on critical thinking, which can be fully answered if the students have a thorough understanding of the concepts taught in the class. This explanation applies to the normal range of students excluding exceptional students.

3)- Is there a different way to convey information without having to be present in the classroom?

This study is based on the performance of the students in two colleges (CCIS and ENG) in a university where the approved mode of teaching and learning is attending face to face classes, supported by the Learning Management System (LMS), and learning resources. The lectures and any other class activity such as labs or tutorials are not recorded for future reference in the normal setting. However, the performance of the students that is affected by their absences in their classes could be improved to some extent if the lectures are recorded and learning resources are uploaded on LMS. However, such video recording cannot compensate face to face classes, especially for the tutorial and lab sessions.

4)- Does online learning affect the outcomes of this research?

The current COVID-19 pandemic has altered the way courses are taught and how the material is presented. The leaning strategy is changed and therefore the effect of absence on students’ performance could be different. For example, all lectures are now recorded and face to face meetings can be conducted online. Hence, another study is required to see whether online absenteeism will imply low academic performance or not. However, from our experience with students, face to face class interactions are very important to motivate students to learn and that cannot fully compensated by recorded lectures.

## 7 Conclusion

This research study showed that student academic performance can be affected by the construction of the timetable. In fact, after introducing our research problem, the state of-the-art related studies have been discussed to point out that this idea has never been introduced. Then, the research questions have been stated before elaborating on the methodology. In the experiment section, the research questions have been addressed and answered using known educational data mining techniques. It has been shown that student academic performance can be affected by the number of absences in addition to some additional factors involving the design of the student schedule. The GPA values were successfully predicted based on the absences, the GPA of the previous semester, and the factors mentioned above. This model can be used by Prince Sultan University or in any other university to alert and/or inform the student about her/his performance based on the timetable design he desires.

Moreover, it has been demonstrated that the absences can be triggered by some main factors related to student’s timetable design including the number of courses, the average number of lectures per day, the average number of breaks per day in addition to the GPA of the previous semester. The later has been added to reinforce the prediction model as both GPA and absences have a strong linear relationship. The absences have successfully been predicted using DN and NO_DN classes. Expectantly, this model allows any university to predict throughout the semester whether the student will be denied from attending the courses or exams. This can help the student to improve his/her situation before the end of the semester. The obtained models can be applied to all colleges in this university. Finally, to improve student academic performance, the timetable should be balanced by avoiding busy or almost free days. This implies a uniform distribution of lectures and breaks throughout the day and the week. This research study can be extended to suggest a new model for timetabling construction. Furthermore, the number of courses should be set according to the students’ previous GPA. If the previous GPA is low, the student should take a minimum number of courses for the current semester. This threshold requires further investigation for future work.

Automated course timetabling has been used to design course timetables that meet certain objectives [35]. It will be interesting to see how automated timetabling can be used to reduce the number of predicted absences in view of the results obtained in this study. Different objectives have been used in automated timetabling, however, to the best of our knowledge these objectives do not take into consideration the predicted number of absences of students. This will be a subject of further investigation in view of the new efficient algorithm for automated timetabling recently developed in [36] which can handle general convex constraints and objectives and can solve large timetables efficiently. For example, the predicted expressions for the number of absences could be used as an objective in automated course timetabling.

## 9 Appendix

### A Appendix: Methodology algorithms

**Algorithm 1**: ROSE: Generating one new artificial case [25]

**Input**: *T*_*n*_: The training set with size *n*; *Y*_*j*_: A class with *j* = 0, 1; *n*_*j*_ < *n*: The number of cases in Class *Y*_*j*_

**Output**: One new Artificial case

1 Select *y* = *Yj*, *j* = 0, 1 with probability $\frac{1}{2}$

2 Select (*x*_*i*_, *y*_*i*_) in *T*_*n*_ such that *y*_*i*_ = *y* with probability $p_{i}=\frac{1}{n_{j}}$

3 Sample *x* from $K_{H_{j}}$ (., *x*_*i*_), with $K_{H_{j}}$ a probability distribution centered at *x*_*i*_ and *H*_*j*_ a covariance matrix.

**Algorithm 2**: SMOTE Pseudocode [26]

**Input**: Number of minority class samples *T*; Amount of SMOTE *N*%; Number of nearest neighbors *k*

**Output**: (*N*/100) * *T* synthetic minority class samples

1 (* If N is less than 100%, randomize the minority class samples as only a random percent of them will be SMOTEd. *) **if** *N* < 100 **then**

2  Randomize the *T* minority class samples

3  *T* = (*N*/100) * *T*

4  *N* = 100

5 *N* = (*int*)(*N*/100) (* The amount of SMOTE is assumed to be in integral multiples of 100. *)

6 k = Number of nearest neighbors

7 numattrs = Number of attributes of the dataset

8 Sample[][]: array for original minority class samples

9 newindex: keeps a count of number of synthetic samples generated, initialized to 0

10 Synthetic[][]: array for synthetic samples

11 (* Compute k nearest neighbors for each minority class sample only. *)

12 **for** *i* ← 1 ***to*** *T* **do**

13  Compute k nearest neighbors for *i*, and save the indices in the nnarray

14  Populate(N, i, nnarray)

15 Populate(N, i, nnarray) (* Function to generate the synthetic samples. *)

16 **while** *N* ≠ 0 **do**

17  Choose a random number between 1 and k, call it nn. This step chooses one of the k nearest neighbors of *i*? **for** *attr* ← 1 **to** *numattrs* **do**

18   Compute: *dif* = *Sample*[*nnarray*[*nn*]][*attr*] − *Sample*[*i*][*attr*]

19   Compute: *gap* = random number between 0 and 1

20   *Synthetic*[*newindex*][*attr*] = *Sample*[*i*][*attr*] + *gap* * *dif*

21  *newindex* + +

22  *N* = *N* − 1

23 **return** (* *End of Populate*. *)

## Abbreviations

ANOVA: Analysis of Variance
CART: Classification and Regression Tree
CBOW: Continuous Bag of words
CCIS: College of Computer & information Sciences
DM: Data Mining
DN: Denial Notice
ENG: College of Engineering
GPA: Grade Point Average
k-NN: k Nearest Neighbors
LR: Linear Regression
MANOVA: Multivariate analysis of variance
MLP: Multilayer perceptron
N-DN: No Denial Notice
ROC: Receiver Operating Characteristics
RT: Regression Tree
SVR: Support Vector Regression
UCI: University of California Irvine Machine Learning Repository
WEKA: A software containing a collection of machine learning algorithms for data mining tasks
